# Homology Modeling of Dopamine D_2_ and D_3_ Receptors: Molecular Dynamics Refinement and Docking Evaluation

**DOI:** 10.1371/journal.pone.0044316

**Published:** 2012-09-06

**Authors:** Chiara Bianca Maria Platania, Salvatore Salomone, Gian Marco Leggio, Filippo Drago, Claudio Bucolo

**Affiliations:** Department of Clinical and Molecular Biomedicine, Section of Pharmacology and Biochemistry, Catania University, Catania, Italy; University of Michigan, United States of America

## Abstract

Dopamine (DA) receptors, a class of G-protein coupled receptors (GPCRs), have been targeted for drug development for the treatment of neurological, psychiatric and ocular disorders. The lack of structural information about GPCRs and their ligand complexes has prompted the development of homology models of these proteins aimed at structure-based drug design. Crystal structure of human dopamine D_3_ (hD_3_) receptor has been recently solved. Based on the hD_3_ receptor crystal structure we generated dopamine D_2_ and D_3_ receptor models and refined them with molecular dynamics (MD) protocol. Refined structures, obtained from the MD simulations in membrane environment, were subsequently used in molecular docking studies in order to investigate potential sites of interaction. The structure of hD_3_ and hD_2L_ receptors was differentiated by means of MD simulations and D_3_ selective ligands were discriminated, in terms of binding energy, by docking calculation. Robust correlation of computed and experimental K_i_ was obtained for hD_3_ and hD_2L_ receptor ligands. In conclusion, the present computational approach seems suitable to build and refine structure models of homologous dopamine receptors that may be of value for structure-based drug discovery of selective dopaminergic ligands.

## Introduction

The dopaminergic systems in the central nervous system (CNS) have been extensively studied over the past 50 years [1]. Dopamine exerts its action through five distinct G-protein coupled receptors (D_1–5_ receptors), grouped in two classes, D_1_-like and D_2_-like receptors, that differ in their signal transduction, binding profile and physiological effects [1]. D_1_-like receptors (D_1_ and D_5_) are principally coupled to stimulatory G_s_-proteins and enhance the activity of adenylyl cyclase (AC), whereas D_2_-like receptors (D_2_, D_3_, and D_4_) are primarily coupled to inhibitory G_i_-proteins and suppress the activity of AC [1].

Alternative splicing of D_2_ receptor mRNA leads to generation of two isoforms: D_2_ short (D_2S_) and D_2_ long (D_2L_), which have been associated (though not exclusively) with presynaptic and postsynaptic populations of D_2_ receptors, respectively [2]. The difference between these two splicing isoforms is represented by 29 amino acid residues in the III intracellular loop (3ICL), involved in the G protein coupling. The D_2S_ is mainly considered as a presynaptic receptor, whereas, the D_2L_ as a postsynaptic receptor [2], like the D_3_ [3]. However, it has been suggested that D_3_, in addition to the classical postsynaptic location, is also localized in the presynapse, where it modulates dopamine release and synthesis [4], [5]. D_2_ and D_3_ receptors display a high degree of sequence homology and share the putative binding site for dopamine and synthetic ligands at the interface of transmembrane helices [6]. D_2_ and D_3_ receptors also share the signal-transduction mechanism, though under certain conditions the latter may exert a weaker stimulation of effectors like AC [7], [8]. Several pathological conditions such as schizophrenia, Parkinson’s disease, Tourette’s syndrome, and hyperprolactinemia have been linked to a dysregulation of dopaminergic transmission [1]. Furthermore, D_2_ and D_3_ receptor have been implicated as potential target for drug development in ocular diseases such as glaucoma [9], [10], [11], [12], [13], [14], [15]. D_2_-like receptors represent the most relevant class in the pathophysiology of neurological and psychiatric disorders. However, while D_2_ receptor is considered the principal target to control the positive symptoms of schizophrenia, none of antipsychotics approved so far discriminates D_2_ from D_3_ receptors; on the other hand, the functional significance of D_4_ receptor largely remains to be defined.

Human dopamine D_2_ receptor (hD_2_) and hD_3_ are highly homologous [16], sharing 78% of sequence identity in the transmembrane domains [17], [18], including the binding site [19]. This sequence identity has introduced difficulties in the design of selective ligands. However, in the past two decades, medicinal chemists have succeeded, by using ligand-based approaches, in developing selective agonists such as aminotetralins: 7-hydroxy-2-dipropylaminotetralin (7-OH-DPAT) [20], trans-7-hydroxy-2-[N-propyl-N(3′-iodo-2′-propenyl)amino]tetratalin (7-OH-PIPAT) [21], [22] and rotigotine [23], [24]. Because the pharmacokinetic profile of 7-OH-DPAT was unsatisfactory, a bioisosteric replacement of the hydroxyphenyl group was carried out [25], leading to ligands selective for D_3_ over D_2_ subtype: quinpirole and pramipexole [26]. More recently a compound with the pyrazole moiety of quinpirole, FAUC 329, was found to selectively activate D_3_ receptor [K_i_ = 4.3 nM] over D_2_ receptor and it has a partial agonist activity (52% compared to quinpirole) [27]. Other drug design studies were carried out successfully by Lopez et al [28] who reported benzolactam derivatives with distinct selectivity against D_3_ and D_2_ receptors; functionalized benzolactam compounds were reported to have D_3_ dopaminergic agonism [29]. Recently, Tschammer et al [30] synthesized heterocyclic dopamine surrogates, among which one compound (biphenylcarboamide (S)-5a) has a very high affinity (27 pM) at the D_3_ receptor and high selectivity over D_2_ subtype.

The crystal structure of hD_3_ has been solved [31] and identified as a powerful tool for structure-based drug discovery of selective dopaminergic D_2_-like ligands [32]. This crystallized receptor is a hD_3_-lysozyme chimera, where the 3ICL is replaced by the lysozime protein; moreover, the receptor bears the mutation Leu119Trp in order to increase the thermal stability of the system. Recently, the determination of the crystal structure of hD_3_ receptor and subsequent efforts in molecular modeling led to successful prediction of the pose of eticlopride in complex with a refined homology model of D_3_ receptor [33]. Kortagere et al [34] analyzed in 2011 the binding mode of preferential D_3_ ligands by means of site-directed mutagenesis and homology modeling studies (template structure 2RH1); these authors identified Ser 192 of V helix as an important site of interaction for the activation of D_3_ receptor. Ser 192 belongs to a cluster of three serine residues (Ser 192, Ser 193, Ser 196); thus we have carefully looked at these residues, and their homologous (Ser 193, Ser 194, Ser 197) in hD_2L_ subtype, in our docking protocol. The subtype selectivity of D_2_-like ligands had been also studied before, by Wang et al [35], in the absence of structural information on D_3_ and D_2_ receptors, by a mixed structure-based (homology modeling using β_2_-adrenergic and rhodopsin receptors, molecular dynamics of haloperidol-receptor complexes) and ligand-based approach (3D-QSAR). These authors, however, did not carry out docking calculations. No study published so far has used a total structure-based approach for modeling ligand interactions with the hD_3_ and hD_2L_. In the present study we aimed at building and validating homology models of hD_3_ and hD_2L_ receptors using the hD_3_ receptor structure (3PBL) as template. Furthermore, in order to better discriminate their structural difference as well as selective ligands, we have carried out a structural optimization by molecular dynamics (MD) simulations of these two receptors for 3 ns in an explicit palmitoyl-oleoyl-phosphatidyl-choline (POPC) bilayer, that mimics the plasma membrane lipid environment, reaching a structural differentiation of these homologous receptors. The short-term MD simulations were adequate to obtain optimized structures of hD_3_ and hD_2L_ receptors, because of the high homology and sequence identity between target and template proteins. We have validated these optimized structures using a total structure-based approach by molecular docking calculations that are extremely influenced by the reliability of receptor structure. The validation of optimized structure models was successful, giving good correlation between experimental and predicted K_i_ of agonists.

## Methods

### Homology Modeling

The retrieved (Swiss-Prot) protein sequences of hD_3_ and hD_2L_ receptors are respectively: P35462.2 and P14416.2. Homology models of hD_3_ and hD_2L_ receptors were obtained by the Automated Modeling tool of Swiss Model web service http://swissmodel.expasy.org/ [36], [37] using the crystal structure of the human D_3_ dopaminergic receptor-lysozyme chimera (Protein Data Bank-code 3PBL) in complex with the antagonist eticlopride as template. N-terminals of receptors were not modeled, because we focused on the binding pocket. Moreover the structure of N-terminal of hD3 was not solved by Chien et al [26]. The terminal residues Tyr 32 in hD3 and Tyr 37 in hD_2L_ were blocked in the homology models by acetylation. The hD_3_ model was validated by docking eticlopride in the binding pocket. The model validation was carried out using two different molecular docking software (the docking protocol is reported in the Docking section): Autodock Vina (Vina) and Autodock 4.2 (AD4.2).

### Molecular Dynamics

Homology models of dopaminergic receptors were embedded in a pre-equilibrated POPC bilayer. Then, the systems were hydrated with TIP3P water molecules, and neutralized adding NaCl up to 150 mM. CHARMM 27 parameters were assigned to all molecules. Disulfide bridges of hD_3_ were parameterized by involving the following residues: Cys 103-Cys 181 connecting the III helix with the II extracellular loop (2ECL) and Cys 355-Cys 358 in the 3ECL. In the hD_2L_ model we parameterized the conserved disulfide bridge between the III helix and 2ECL involving the Cys 109-Cys 187 residues. The system preparation processes (building of bilayer, embedding of the proteins into the membrane, hydration and neutralization) were done using VMD v1.8.7 [38]. Before MD simulations the systems were equilibrated as follows: i) MD of lipid tails for 50 ps (time-step = 1 fs) while protein, water, ions and lipid head groups were kept fixed; ii) equilibration for 100 ps (time-step = 1fs) of water-ions-lipids, while proteins were kept fixed by applying harmonic constraints; iii) 500 ps (time step = 1 fs) of system equilibration, with no constraints applied to molecules. After the described steps of equilibration, 3 ns of MD simulation were carried out with time-step of 2 fs, collecting trajectory data every 10 ps. The SHAKE algorithm, which constraints the hydrogen-heavy atom bonds.was applied. Equilibration steps and simulations were carried out using NAMD v2.7 [39]. Langevin dynamics and piston were used to maintain constant temperature (300 K) and pressure (1 atm) during simulation. The area per lipid was maintained constant, after the equilibration steps (NPAT ensemble). The particle number of systems was 83242 for hD_3_-lipids-water-ions and 83429 for hD_2L_ in membrane. Periodic Boundary Conditions (PBC) and Particle Mesh Ewalds (PME) method [40] were used to treat long-term electrostatics (time-step of 4 fs). The cut-off at 10 Å was applied to Van der Waals and coulombic interactions and switching functions started at 9 Å. First stage minimization was performed using the steepest descent algorithm whereas the conjugate gradient was used during production runs.

### Docking and Virtual Screening

We carried out two different molecular docking studies using Vina and AD4.2 software. Vina [41] is an accurate algorithm faster than AD4.2; for this reason it was used for docking calculation of a large group of D_2_-like ligands and for virtual screening study. AD 4.2 [42] provided the best prediction of pose of eticlopride in the hD_3_ homology model, thus we have chosen it for accurate docking calculation such as prediction of K_i_ of well-known D_2_-like agonists docked into the refined homology models of hD_3_ and hD_2L_ receptors. File preparation for AD4.2 docking calculations was carried out using the AutodockTool (ADT), a free graphics user interface (GUI) of MGL-tools.

The search space for all docking calculations included the orthosteric binding pocket individuated by eticlopride in 3PBL, the allosteric binding pocket reported by Chien et al [31] and the extracellular domain of receptors. An high exhaustiveness, 32, was used in Vina calculation because the search space applied to hD_3_ and hD_2L_ receptor is relatively wide. In calculations carried out with AD4.2 we chose, as search algorithm, the time-consuming Lamarkian genetic algorithm (GA), that generated the best docking results for eticlopride in hD_3_ homology model. Hundred iterations of GA with 2,500,000 energy evaluations per run were carried out. Population size was set to 150 and a maximum of 27,000 generations per run was carried out, followed by automatic clusterization of poses. Top scored (lowest energy) and more populated poses with orthosteric binding, as reported by Kortagere et al [34],were selected for analysis of ligand-protein interactions using the GUI ADT. AD 4.2 uses a semi-empirical free energy function and a charge-based method for desolvation contributes; the free energy function was calibrated using a set of 188 structurally known ligand-complexes with experimentally determined binding constants [43]. The binding energy of ligand poses (Kcal/mol) is the sum of intermolecular energy, internal energy of the ligand and torsional free energy minus the unbound-system energy (see in Supporting Information S1 about the calculation of K_i_ from AD4.2 binding energy values and Supporting Information S2 for ligand poses and optimized structure of receptors).

### Ligand Dataset

**Figure 1:**
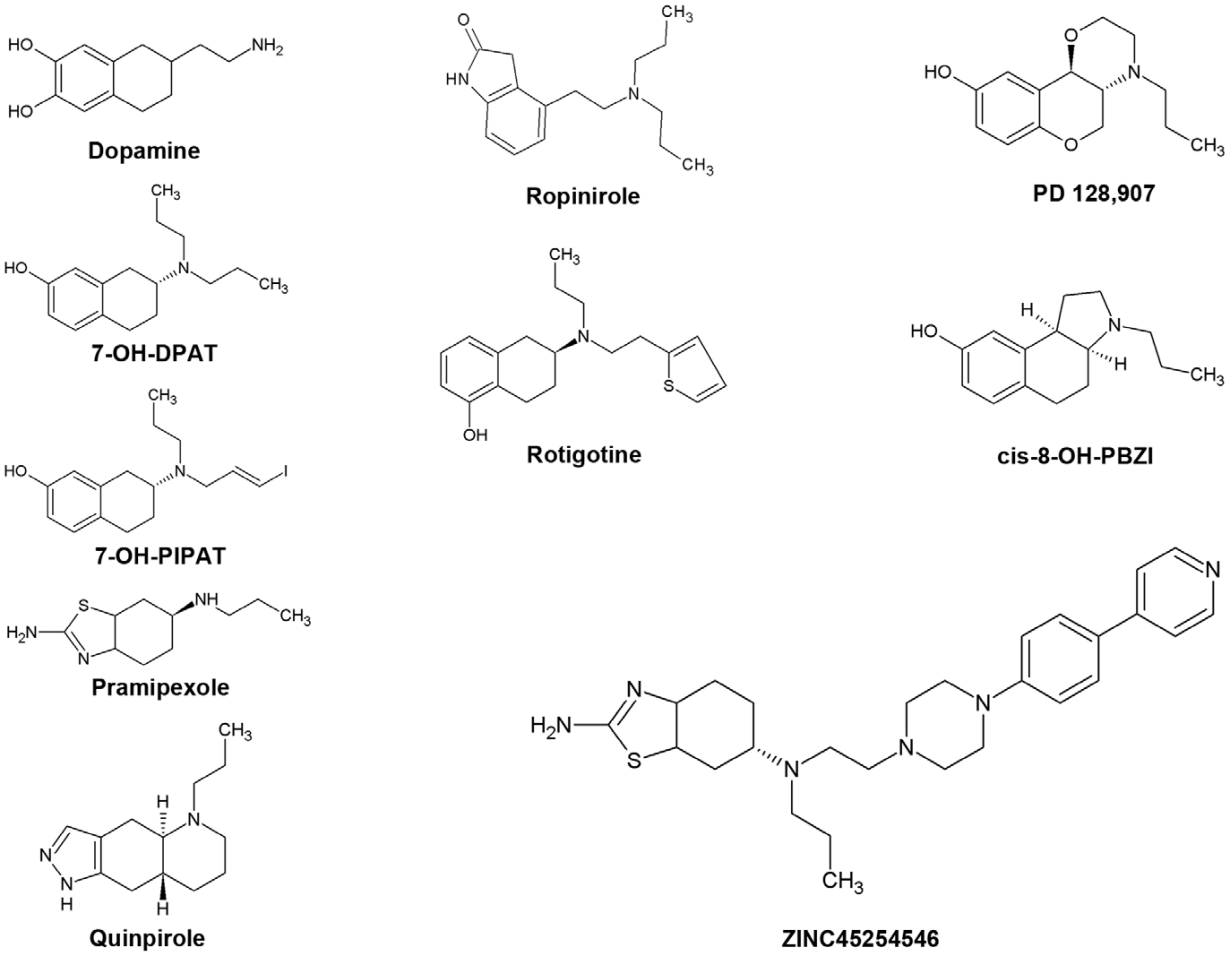
D_2_-like agonists.

**Figure 2:**
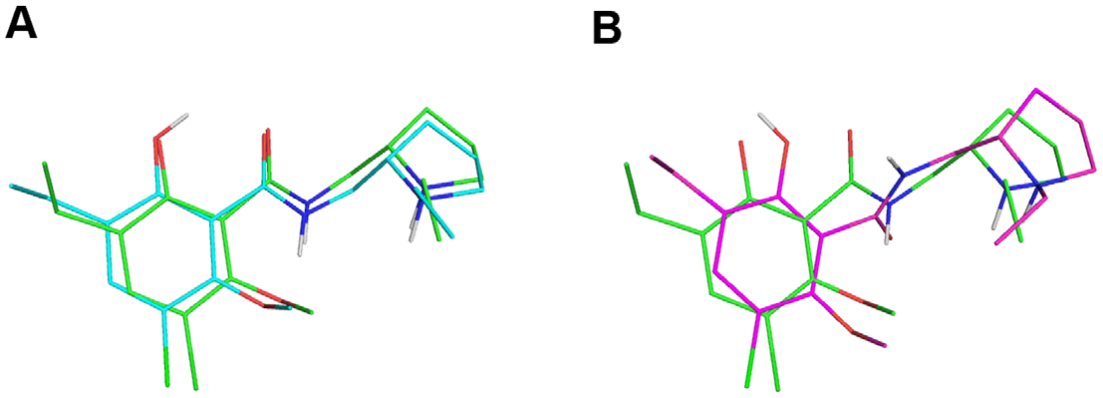
Re-docking eticlopride. Superimposition of eticlopride re-docked with AD4.2 (cyan lines, A) and with Vina (magenta lines, B) toward eticlopride in complex with hD_3_ in the crystal structure 3PBL (green lines).

**Figure 3:**
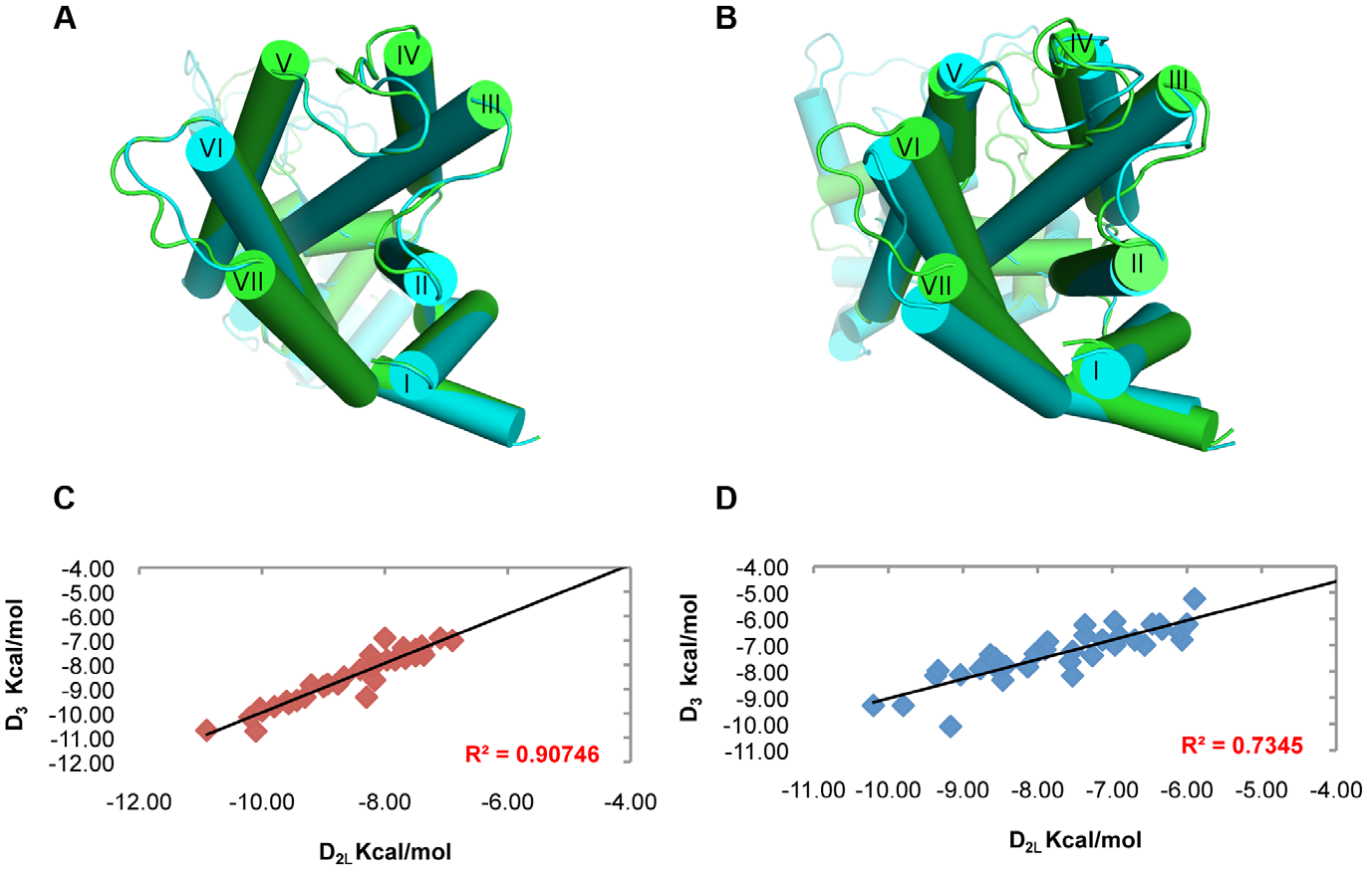
Structure differentiation of hD_3_ and hD_2L_ receptors simulated in membrane. (A) Superimposition of hD_3_ (green cartoon) and hD_2_ (cyan cartoon) homology models before the refinement with simulation in membrane. (B) structural alignment of hD_3_ (green cartoon) and hD_2_ (cyan cartoon) receptors after 3 ns of MD simulation in membrane. (C) high correlation of hD_3_ and hD_2_ binding energies (Autodock Vina) of D_2_-like ligands from homology models without MD refinement. (D) low correlation of hD_3_ and hD_2_ binding energies (Autodock Vina) of D_2_-like ligands after MD refinement.

**Figure 4:**
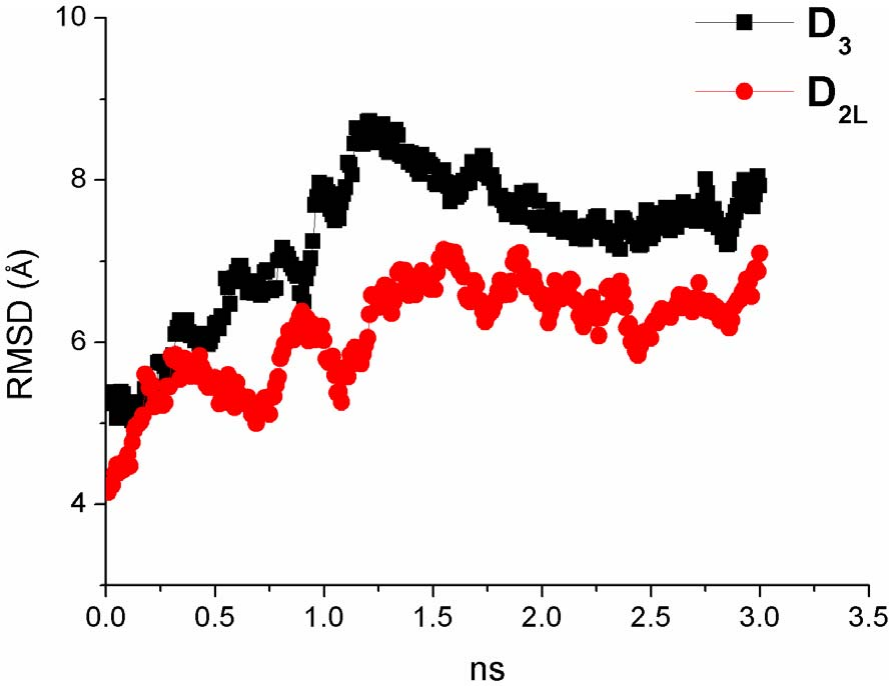
Analysis of Root Mean Square Deviation of Cα atoms during molecular dynamics simulation. RMSD respect to the starting structures, homology models, of hD_3_ (black squares) and hD_2L_ (red circles) receptors.

**Figure 5:**
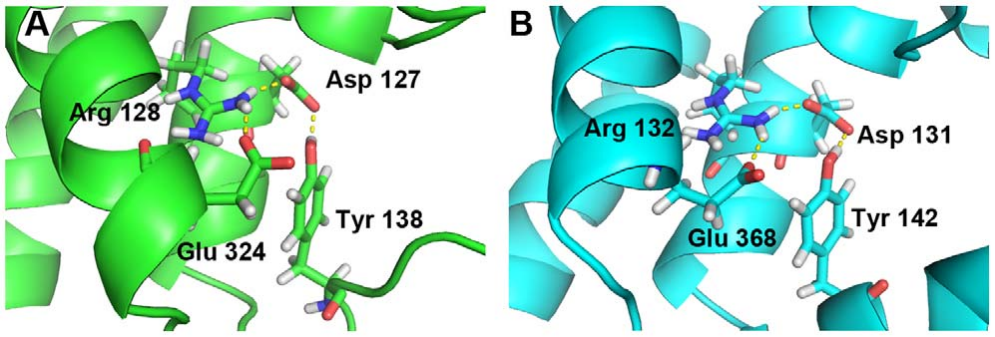
Ionic-look, structural marker of inactive state of G-protein Coupled Receptors. (A) hD_3_ and (B) hD_2L_ receptor.

**Figure 6:**
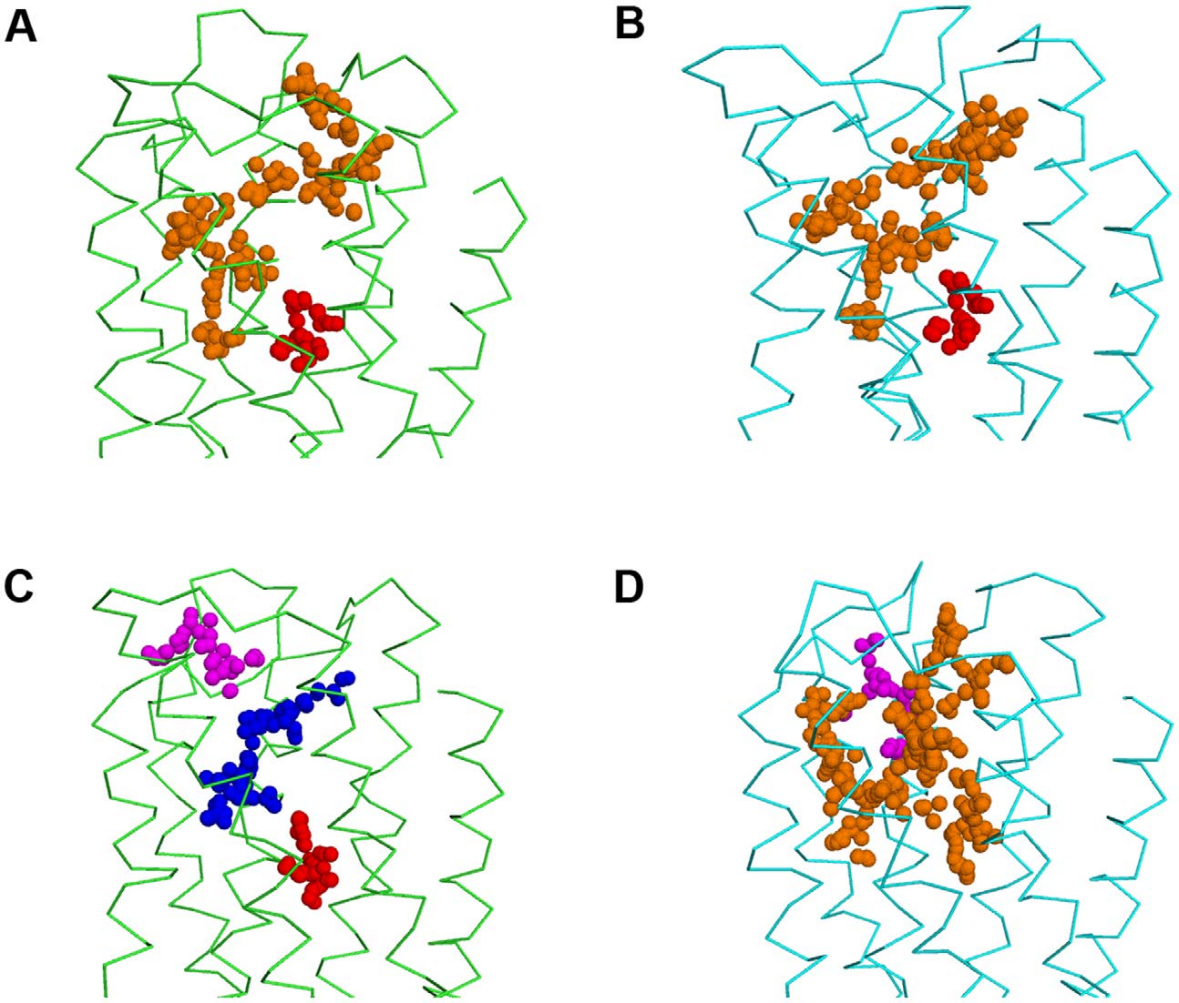
Evolution of binding pockets of hD_3_ and hD_2L_ receptor after model refinement. Pockets generated by Fpocket server are represented as colored clusters of spheres. Left panels represent hD_3_ (green ribbons) and right panels represent hD_2L_ (cyan ribbons), before (A, B) and after (C, D) MD simulations. The red circles target the orthosteric binding pocket whereas the black circles highlight the allosteric binding pocket.

**Figure 7:**
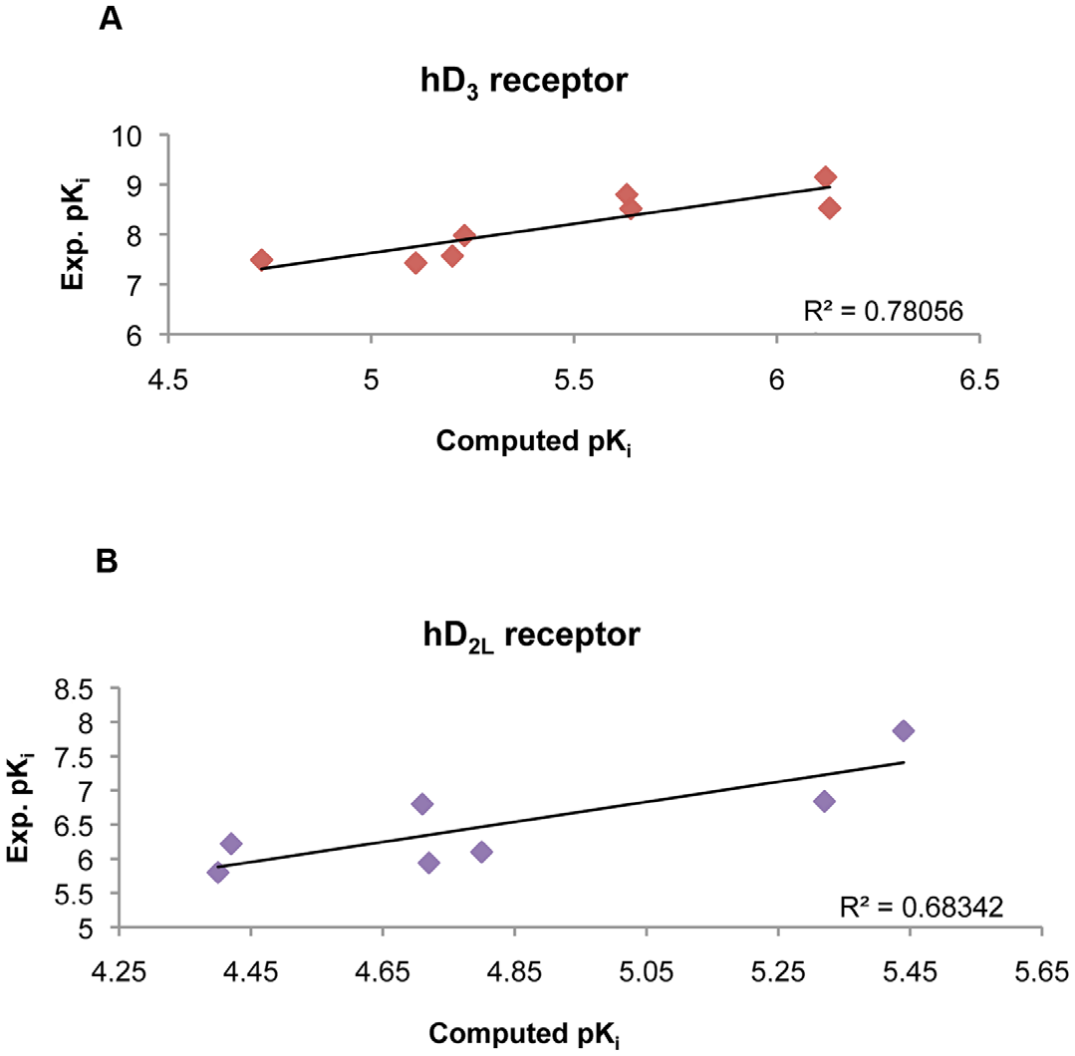
Correlation of predicted pK_i_ and experimental pK_i_ values. Plots of D_3_ preferring agonists docked toward hD_3_ (A) and hD_2L_ (B) receptors: a. dopamine; b. 7-OH-DPAT; c. 7-OH-PIPAT; d. pramipexole; e. quinpirole; f. ropinirole; g. rotigotine; h. PD 128,907; i. cis-8-OH-PBZI; j. ZINC45254546.

Structure files of ligands were retrieved from PubChem [44], ZINC database [45], and, when not available there, from PRODRG web service (http://davapc1.bioch.dundee.ac.uk/prodrg/), as.mol2 files [46]. Whenever a conversion of file format was necessary it was done by Open Babel [47]. Protonation state of ligands was assigned at pH = 7.4. Three replicas of dockings were carried before and after MD simulations in order to assess the structure differentiation of homology model simulated in membrane. The following ligands were used in fast docking calculations with Vina: r-7-OH-DPAT, s-7-OH-DPAT, r-7-OH-PIPAT, s-7-OH-PIPAT, bromocriptine, lergotrile, lisuride, pergolide, cianergoline, cabergoline, SDZ-GLC-756, PD128907, pramipexole, rotigotine, ropinirole, eticlopride, U99194A, Ru24213, GR103691, r-GSK89472, s-GSK89472, s-nafadotride, NGB2904, PG01037, PNU177864, SB-269-652, S33084, SB277011A, S14297, S17777 and compounds of the USC series from Ortega et al [29] (USC-A401, USC-B401, USC-H401, USC-I401, USC-K401, USC-M401). The D_3_ agonists, represented in Figure 1, r-7-OH-DPAT, r-7-OH-PIPAT, pramipexole, ropinirole, rotigotine, quinpirole, dopamine, PD128907 and cis-8-OH-PBZI (cis-8-hydroxy-3-(n-propyl)1,2,3a.4,5,9b-hexahydro-1H-benz[e]indole) were docked with AD4.2 into the hD_3_ and hD_2L_ receptors optimized by MD; the predicted K_i_ values were correlated to the experimental ones. Eighty nine compounds, retrieved from ZINC database, were used to build a small focused drug-like database of ligands (according to the Lipinski’s rule of five and similar at 70% to pramipexole); they were docked with Vina into hD_3_ and hD_2L_ refined receptors. Structural alignments of proteins and figures were done with the molecular visualization software Open PyMOL. All software utilized in our study were open source or under free of charge academic license. Computational hours were provided by the GRID service “Consorzio Cometa” [http://www.consorzio-cometa.it/].

## Results

### Homology Modeling

We built the homology models of hD_3_ and hD_2L_ receptors. Two disulfide bridges were modeled in hD_3_ receptor according to the crystal structure 3PBL [31], the canonical one that connect the 2ECL with the III helix and the disulfide bridge in the 3ECL involving residues Cys 355 and Cys 358. In hD_2L_ receptor only the conserved disulfide bridge was modeled, because we considered that a single residue of distance between the two conserved cysteine residues (Cys 399 and Cys 401) may lead to unstable disulfide bond. Validation for the hD_3_ model, by docking eticlopride with Vina and AD4.2 was performed. Both software were able to reproduce the eticlopride conformation in the binding pocket; AD4.2 gave the lowest root mean square deviation (RMSD, 0.4 Å) and better reproduced the internal H-bonds (Figure 2A), compared to VINA (Figure 2B), that gave 0.6 Å RMSD for re-docked eticlopride. We have evaluated the similarity of hD_3_ and hD_2L_ homology models by means of structural alignment. The tridimensional alignment revealed that the two homology models did not differ in transmembrane core structure (Figure 3A), as expected from their high sequence identity; furthermore, RMSD between the two aligned GPCRs was very low (0.033 Å). We have, further, analyzed the structural similarity and capacity of discrimination of active D_2_-like ligands by fast docking calculations, with the Vina docking software. The structure similarity was reflected by the high correlation (R^2^ = 0.91, Figure 3C) of predicted binding energy of D_2_-like ligands docked into the homology models of hD_3_ and hD_2L_. Thus, these two homology models do not seem useful, without a structural refinement, for virtual screening directed at the recognition of selective ligands.

### Molecular Dynamics

We have simulated for 3 ns the hD_3_ and hD_2L_ homology models in a water-membrane environment that reproduces the biological milieu where these two GPCRs are located, to further discriminate their structural difference. By reporting the RMSD of protein structure from the starting homology model, both receptors differentiate in structure and reach a relative stable conformational minimum roughly after 1.25 ns (Figure 4). Total energy (E_tot_) and potential energy (E_p_) of systems are constant during the MD simulation (Supporting Information S1) and energy values of D_3_ receptor are slightly lower compared to the energy of D_2L_ subtype. We stopped simulations at 3 ns because we reached stable local minima and distinct conformations for hD_3_ and hD_2L_ receptors. Longer simulations (over 30 ns) might reveal other local minima and further characterize the conformational space of these receptors; this goal, however, is beyond the aim of our study. GPCRs are in equilibrium between active and inactive conformation, and, as far as the inactive conformation is concerned, a structural marker, the “ionic lock” was described in several studies [48], [49], [50], [51] and was also revealed in the crystal structure of eticlopride-hD_3_ complex (3PBL) [31]. This ionic lock involves, four conserved residues, Arg128-Asp127-Glu324-Tyr138 in hD_3_ (Figure 5A), and Arg132-Asp131-Glu368-Tyr142 in hD_2L_ receptor (Figure 5B), respectively. The salt-bridges that constitute the ionic lock are retained during the 3 ns of simulation. We can assume that the conformation of receptors, that reached the relative minimum, describes the inactive state. The superimposition of the simulated hD_3_ and hD_2L_ receptors confirmed the structural deviation of receptors in membrane, as the RMSD was 1.63 Å (Figure 3B). The differentiation of the two homologous receptors was further strengthen by the lower correlation (R^2^ = 0.74) of binding energies of D_2_-like ligands docked, with VINA, into hD_3_ and hD_2L_ optimized structures (Figure 3D). We have measured the Cα deviation of residues belonging to the orthosteric binding pocket of receptors in order to further characterize the structural modification of hD_3_ and hD_2L_ induced by the membrane environment. The deviations of these residues, comparing the initial homology models with the refined structures are reported in Table 1. The residues of binding pocket of hD_2L_ receptor deviated from starting model more than residues of hD_3_ subtype (Table 1). The V helix of hD_2L_ receptor had the greater deviation than other helices after the simulation (Supporting Information S1), involving the extracellular and intracellular side (transversal to the plane of the membrane). The VI and VII helices deviated mostly in the extracellular side and the greater deviation is shown for the VII helix (Supporting Information S1). Within the seven helices of hD_2L_ receptor, only IV helix had a major transversal deviation and a sensible deviation along the z-axis of membrane (Supporting Information S1). Furthermore, the binding pocket of hD_3_ receptor was also remodeled in membrane, because there were major structural deviations involving the residues of V helix (Ser 192, Ser 193, Ser 196), VI helix (His 349) and VII helix (Tyr 375) (Tables 1 and Supporting Information S1). We further characterized the binding pocket of hD_3_ and hD_2L_, before and after refining with MD simulations, by using the web service fpocket http://fpocket.sourceforge.net/ [52]. Fpocket generates clusters of spheres to describe each pocket of a given protein; in Figure 6 we have assigned different colors to pockets of hD_3_ and hD_2L_ receptors, before and after optimization. Before simulation in membrane, the binding pockets of the two receptors were very similar in shape and dimension. After simulation, the pocket of hD_3_ became smaller than that of hD_2L_ and divided in three pockets (Figure 6C); the one in blue includes the orthosteric and the allosteric pockets, the one in magenta is surrounded by the extracellular loops, and the deepest and smallest pocket is colored in red. In docking calculations, we did not find poses in the red pocket, that was occupied by water molecules during MD simulation (data not shown). The pocket of hD_2L_ after simulation became bigger than that of D_3_ subtype (Figure 6B and 6D). The hD_2L_ receptor after simulation shows a big pocket (orange spheres) and a smaller pocket (magenta) located along the big one, between the III and IV helices. After simulation the red pocket of hD_2L_ appears included within the orange one (Figure 6B and 6D). The optimized structures of hD_3_ and hD_2L_ used for analysis and docking calculations were extracted randomly from one of the last frames of simulations that characterize the relative conformational equilibrium, by considering as equivalent frames belonging to the same local minimum. To confirm this assumption we randomly selected one additional frame from each local minimum of the hD_3_ and hD_2L_ MD simulations. These two additional frames resulted equivalent to the previous, because, when carrying out docking of pramipexole superimposable results were obtained both in terms of binding energy (Table 2, values in brackets) and poses (data not shown). We did not carried out a clusterization of trajectories because we have reached one local minimum in each simulation. Furthermore, as reported by Yap et al [53] clusterization of GPCR trajectories, is not useful for selecting the representative structure to be used in docking calculation.

**Table 1:** Deviations of Cα of residues belonging to the orthosteric binding pocket of optimized receptors in comparison with the starting models.

hD_3_		hD_2L_	
Residue	Cα deviation (Å)	Residue	Cα deviation (Å)
Asp 110 (III helix)	0.3	Asp 114 (III helix)	1.3
Ser 192 (V helix)	0.9	Ser 193 (V helix)	1.3
Ser 193 (V helix)	0.9	Ser 194 (V helix)	1.0
Ser 196 (V helix)	1.3	Ser 197 (V helix)	3.2
Trp 342 (VI helix)	0.3	Trp 386 (VI helix)	1.5
Phe 345 (VI helix)	0.3	Phe 389 (VI helix)	1.8
Phe 346 (VI helix)	0.3	Phe 390 (VI helix)	0.9
His 349 (VI helix)	0.6	His 393 (VI helix)	1.8
Tyr 375 (VII helix)	1.2	Tyr 416 (VII helix)	0.9

**Table 2:** Predicted binding energy (Autodock 4.2) of D_3_ agonists towards hD_3_ and hD_2_ receptors. Experimental K_i_ (exp. K_i_) with respective references are also shown.

D_3_ agonist [reference]	hD_3_ E_binding_ (kcal/mol)	hD_2_ E_binding_ (kcal/mol)	hD_3_ exp. Ki (nM)	hD_2_ exp. Ki (nM)
Dopamine	−6.5	−6.0	32.5(1)	598(1)
r-7-OH-DPAT [61]	−7.7	−6.4	1.58	158
r-7-OH-PIPAT [19]	−8.4	−7.3	2.9(2)	142(2)
Pramipexole [62]	−7.1	−6.6	10.5	790
Pramipexole(3)	(−7.1)	(−6.4)		
Ropinirole [62]	−7.0	−6.4	37.2	933
Rotigotine [63]	−8.4	−7.4	0.71	13.5
Quinpirole [64]	−7.6	−6.6	39	1402
PD 128907 [65]	−7.7	−6.0	3.1	1573
cis-8-OH-PBZI [66]	−7.1	ND	27.4	ND

(1) Average value from PDSP database: http://pdsp.med.unc.edu/indexR.html.

(2) The K_i_ is reported for the racemic 7-OH-PIPAT.

(3) Pramipexole re-docked in two other frames of hD_3_ and hD_2L_ receptor; see also text.

### Docking

We validated the optimized structures of hD_3_ and hD_2L_ receptors by docking D_3_–preferring receptor agonists into receptor binding pockets using AD 4.2 docking software, which provided the best result of eticlopride pose prediction in the hD_3_ homology model. Binding energy of agonists docked in hD_3_ and hD_2L_ receptors correlates with their higher affinity for the D_3_ subtype (Table 2), consistent with more polar contacts of ligands docked into D_3_ receptor compared to ligands docked into the D_2L_ subtype (Table 3). The experimental pK_i_ values (retrieved from http://pdsp.med.unc.edu/free access database) of agonists were compared with the predicted values (Figure 7, see also Supporting Information S1) obtaining a good correlation as indicated by Pearson coefficients relative to hD_3_ and hD_2L_ receptors equal to 0.88 and 0.83 respectively (p<0.005). Linear regression coefficients however were low (Figure 7), due to the limitations of AD4.2 in predicting absolute values of K_i_, as reported by Lape et al [54] and by Yap et al [55]. Another explanation to the mentioned issue might be related to the heterogeneity in K_i_ determination assays. Quinpirole was not included in the regression analysis because it was an outlier, even though its predicted binding energies for hD_3_ and hD_2L_ correlate with the higher affinity toward the D_3_ subtype. Quinpirole is a bioisoster of DPAT, among other ligands included in the regression model (Figure 1), with a tricyclic structure where the hydroxyphenyl group is substituted with a pyrazolic group. On the contrary, PD-128907, a tricyclic compound with the hydroxyphenyl group, fits in the regression model of pK_i_ for hD_3_ and hD_2L_ receptor. Another tricyclic compound included in the regression model is cis-8-OH-PBZI (PBZI), which retains the position of hydroxyl and amine groups of 7-OH-DPAT. The affinity of PBZI was determined for D_2S_, D_3_ and D_4_ receptors but not for D_2L_ receptor, therefore we did not include it in the regression model for hD_2L_ receptor. Recently, PBZI was found to not induce tolerance and slow response termination, in comparison to known agonists such as 7-OH-DPAT and pramipexole [56]. Comparing the tricyclic structures of PD-128907, PBZI and quinpirole, this latter might behave as an outlier in the chemical space, due to the substitution of the hydroxyphenyl moiety with the pyrazol condensed group.

**Table 3:** Ligand protein-interaction of D_3_–preferring receptor agonists docked with AD4.2.

Ligands	hD_3_		hD_2L_	
	Hydrogen bonds-polar contacts	Hydrophobiccontacts	Hydrogen bonds-polar contacts	Hydrophobic contacts
Dopamine	Asp 110, Thr 115, Ser 192,Ser 196.	Ile 183, Phe 345, His 349.	Asp 114, Ser 194	Val 115, His 393, Phe 389, Phe 390.
r-7-OH-DPAT	Asp 110, Ser 192, Ser 196,Thr 115.	Ile 183, Phe 345, His 349.	Asp 114,, Ser 193.	Val 111, Phe 110, Ile 184, Phe 390.
r-7-OH-PIPAT	Asp 110, Val 111 (C = Oof peptide bond), Thr 115,Ser 192.	Val 111, Val 107, Ile 183,Trp 342, Phe 345, His 349.	Asp 114, Val 190 (C = O of peptide bond), Ser 193.	Val 111, Phe 110, Ile 184, Phe 390.
Pramipexole	Asp 110, Thr 115, Ser 192,Ser 196.	Val 111, Trp 342, Phe 345,Thr 369.	Asp 114, Val 190 (C = O of peptide bond), Ser 194.	Phe 110, Val 111, Phe 390, His 393.
Ropinirole	Asp 110, Ser 192	Val 189, Trp 342, Phe 345,His 349, Tyr 373	Asp 114, Ser 193.	Val 111, Phe 110, Val 115, Phe 390, His 393
Rotigotine	Asp 110, Ser 192.	Val 107, Phe 106, Phe 345,Phe 346, His 349	Asp 114	Phe 110, Val 111, Val 115, Ile 184, Phe 390, His 393
Quinpirole	Asp 110, Ser 192	Val 111, Ile 183, Trp 342,Phe 345, Thr 369, Tyr 373.	Asp 114	Val 115, Trp 386, Phe 389, Gly 415, Tyr 416.
PD128907	Asp 110, Ser 192	Val 111, Ile 183, Phe 188,Trp 342, Phe 345, Phe 346,Thr 369, Tyr 373.	Asp 114	Val 111, Phe 389, His 393.
cis-8-OH-PBZI	Asp 110, Ser 192, Ser 196,Thr 115	Val 111, Ile 183, Trp 342,Phe 346, Tyr 373, Thr 369.	*ND	*ND

* ND = Not Determined.

Residues involved in H-Bonds are underlined.

### Virtual Screening

**Figure 8:**
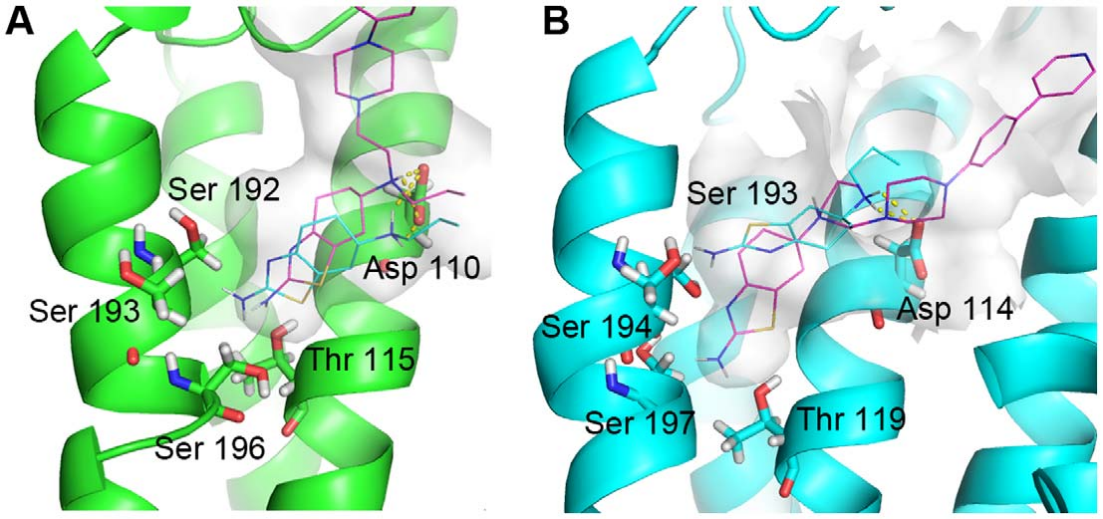
Virtual screening. Pose of pramipexole (cyan lines) and compound ZINC45254546 (magenta lines, see also text) docked into hD_3_ (A) and hD_2L_ (B) optimized receptor structures. H-bonds with Aspartate conserved residues are represented with yellow dashes.

Pramipexole is a selective D_3_ agonist (D_2_/D_3_ = 75.5) indicated in the treatment of early-stage Parkinson disease. This agonist was chosen as reference for building a small ligands database (89 molecules), where drug-like compounds are 70% similar to pramipexole. We carried out a virtual screening by docking these ligands into the refined hD_3_ and hD_2L_ models. The top scored compound is a novel selective D_2_-like agonist synthesized by Ghosh et al [57] (-)-(S)-N6-Propyl-N6-(2-(4-(4-(pyridin-4-yl)phenyl)piperazin-1-yl)-ethyl)-4,5,6,7-tetrahydrobenzo[d]-thiazole-2,6-diamine, deposited in the ZINC database with the name ZINC45254546. This compound is reported to have high affinity towards hD_3_ subtype (D_2L_/D_3_ = 56.5) (Table 4). ZINC45254546 (Figure 1) is an hybrid compound bearing a pramipexole moiety and a piperazin(4-phenyl(4pyridyl)) antioxidant group. This compound was re-docked with AD4.2, into hD_3_ and hD_2L_ receptors. As shown in Figure 8, polar contacts involved aspartate and threonine residues in III helix and the cluster of serine residues in V helix that interact with the pramipexole group. The analysis of pose of ZINC45254546 did not show the H-bond with Asp114 in hD_2L_, which may explain its lower affinity toward the D_2L_ subtype. The piperazin(4-phenyl(4pyridyl)) group interacted with part of the 2ECL in hD_3_ subtype and with residues of II and VII helices in hD_2L_ receptor, that characterize the allosteric pocket. The top 30 compounds (ZINC-db code), docked into hD_3_ and hD_2L_ receptors, are reported in Supporting Information S1.

**Table 4:** Virtual Screening. Top scored compound ZINC45254546.

	hD_3_	hD_2L_
Vina (Kcal/mol)	−8.7	−8.1
AD4.2 (Kcal/mol)	−8.8	−7.98
Exp. Ki (nM)	4.78	270
H-bonds and Polar contacts	Asp 110, Thr 115, Ser 196, Ser 182,	Ser 197, Ser 193, Thr 119
Hydrophobic interactions	Val 111, Ile 183, Phe 345, Phe 346, His 349, Tyr 365,Pro 362,Thr 369.	Leu 94, Val 91, Val 111, Ile 184, Val 115, Phe 198, Phe 389, Phe 390, His 393, Thr 412, Tyr 416.

Residues involved in H-bonds are underlined.

## Discussion

In the present study we have successfully modeled and optimized the structure of two high homologous GPCRs, the hD_3_ and hD_2L_ receptors. The homology modeling is a powerful tool in the prediction of protein structure. The strength of this methodology is related to the sequence identity shared between the target and the template protein: the highest sequence identity determines the best structure model. We built and validated the homology models of hD_3_ and hD_2L_ receptor using the x-ray structure of hD_3_ receptor, a lysozyme-chimera protein. The high sequence identity shared by these two receptors did not allow us to differentiate their homology models that were therefore unsuitable for prediction of binding energies and subtype selectivity of D_2_-like ligands. The high structure similarity of hD_3_ and hD_2L_ arises from the energy minimization process, and represents a weakness in the homology modeling approach. Usually, in homology modeling, the energy optimization of the modeled protein structure is performed by energy minimization *in vacuo*, with some exceptions such as the GPCRRD server http:/zhanglab.ccmb.umich.edu/GPCRRD/. GPCRRD carries out a pipeline of structural optimizations of homology models, with a final MD simulation: Fragment-Guided Molecular Dynamics (FD-MD), which takes into account knowledge-based (H-bonds and positional restraints) and physics-based atomic potentials (AMBER99 forcefield) [58], [59]. So far protein-lipid and protein-water explicit interactions, based on empirical physics-based atomic potentials, are not taken into account by homology modeling software. Thus, we attempted to optimize the structure of the hD_3_ and hD_2L_ models by MD in an explicit water-membrane environment, reaching a local conformational minimum within 3 ns. The MD simulations led to structural adaptation and differentiation of the two receptors in membrane, enabling the prediction of trends of pK_i_ values and the modeling of ligand-protein interactions of D_3_-preferring receptor agonists. Moreover, the refined models were useful in the identification, by a virtual screening approach, of an agonist (ZINC45254546) referred to be selective for D_3_ over D_2_ [57]. Our results are consistent with the findings of Chien et al [26]; the hD_3_ homology model we built was validated by docking eticlopride and by obtaining with AD 4.2 a pose highly similar to the one in the x-ray structure 3PBL. Because the ionic lock, a marker of inactive state described in 3PBL, was retained during MD simulations in both hD_3_ and hD_2L_ receptors, we can assume that refined models represent an inactive state of the receptors. Moreover, we modeled both disulfide bridges solved in 3PBL in hD_3_ model and we modeled just one disulfide bridge, the canonical one, in hD_2L_. We made this choice because the conserved cysteine residues in the 3ECL, Cys 399 and Cys 401, are separated just by one residue Asp 400, leading to a high constrained loop in the case a disulfide bridge is formed. The lack of the accessory disulfide bridge in the 3ECL might have influenced the dynamics of hD_2L_ receptor, leading to the swelling of its binding pocket, in comparison to the hD_3_ which is restrained by two disulfide bridges. Wang et al [60] have predicted the structural differences of hD_3_ and hD_2_ receptors. The homology models of these GPCRs were built in complex with haloperidol (previously aligned to the β_2_-adrenergic inverse agonist s-carazolol), using the crystal structure of β_2_-adrenergic receptor (2RH1); the complexes were subsequently simulated in a POPC bilayer for 1.5 ns. Haloperidol in complex with simulated D_3_ and D_2_ receptors was also used to carry out 3D-QSAR studies using 163 compounds. These authors [35] concluded that the higher affinity of bigger ligands for D_3_ receptor over D_2_ subtype is related to the shape of binding pocket, which is shallower in D_2_ receptor. We found that the binding pocket of hD_3_ receptor, after adapting in the membrane environment, significantly deviates from the initial homology model, becoming smaller and partitioned. The binding pocket of hD_3_ in membrane environment is also smaller than the one of hD_2L_ receptor. We carried out docking calculations rather than 3D-QSAR (ligand-based method) because we considered our refined models highly predictive due to the crystal structure of hD_3_ receptor, used as template for homology modeling. Docking calculations (structure-based method) are strictly related to the reliability of the receptor structure, and we obtained a good correlation of experimental and computed K_i_ values for agonists docked into hD_3_ and hD_2L_ binding sites. Although the prediction of absolute K_i_ values is a difficult task, AD 4.2 was a powerful tool in order to validate homology model of hD_3_ receptor (eticlopride re-docking) as well as to validate the refined models by MD simulations. In fact, the predicted trend of K_i_ values is well correlated (high Pearson coefficients) with the experimental trend. This correlation was carried out with aminotetraline derivatives, a congeneric chemical class that does not include quinpirole. This latter is a preferential D_3_ agonist, but behaved as an outlier in the chemical space of docked ligands, due to the tricyclic structure and the pyrazole moiety. Neverthless, our optimized models were able to predict the affinity of quinpirole higher for D_3_ than for D_2L_ receptor. In conclusion, the computational approach, totally structure-based, adopted in the present study is able to build and refine structure models of homologous dopamine receptors that may be of interest for structure-based drug discovery of selective dopaminergic ligands, potentially useful to treat neurological, psychiatric and ocular disorders.

## Supporting Information

Supporting Information S1 Figure S1: Energy plots of systems. Potential energy (E_pot_) and total energy (E_tot_), of hD_2L_ and hD_3_ receptors. Table S1: Cα deviations of transmembrane helices (TM) of D_3_ and D_2L_ simulated receptors from the starting models. Cα deviation values were determined by structural alignment of each helix of the model and of the optimized structure. Figure S2: Deviation of helices of optimized hD_2L_ receptor (cyan cartoon) respect the starting model (yellow cartoon). The upper side of the figure corresponds to the extracellular side. Table S2: Computed pK_i_ for ligands docked into hD3 and hD2L receptors. Values are reported for ligands inserted in the regressions represented in Figure 7. Figure S3: Superimposition of template (3PBL)-homology model- optimized model of hD_3_ receptor and hD_2L_ receptor. The template structure (green cartoon) is the A chain of hD_3_ receptor crystal structure (3BPL). The cyan cartoon corresponds to the homology model of hD_3_ receptor, the yellow cartoon corresponds to the homology model of hD_2L_ receptor. The optimized models of hD3 and hD2L receptor are respectively the magenta and orange cartoons.
(DOCX)

Supporting Information S2 Supplemental files (.pdb files) contained in the compressed directory File S2 include poses of ligands, shown in Figure 1, docked into hD_3_ and hD_2L_ optimized receptors, whose.pdb files are also included in File S2. All.pdb files can be visualized with Open Pymol. Files named ligand_D2.pdb correspond to poses of ligand docked into hD_2L_ receptor, whereas files named ligand_D3.pdb correspond to poses into hD_3_ receptor. The optimized structure of hD3 and hD2L receptor are named respectively opt_D3_receptor.pdb and opt_D2L_receptor.
(ZIP)
