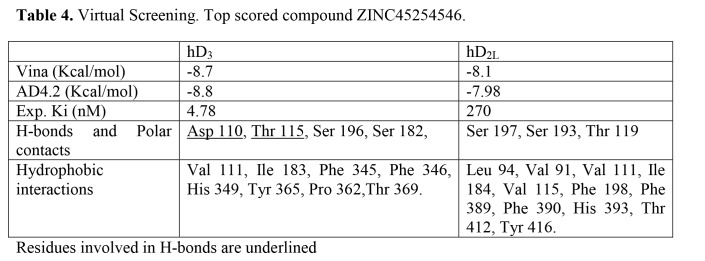# Correction: Homology Modeling of Dopamine D_2_ and D_3_ Receptors: Molecular Dynamics Refinement and Docking Evaluation

**DOI:** 10.1371/annotation/bd61cc74-2a2c-4433-a78d-84fa2310e919

**Published:** 2013-01-16

**Authors:** Chiara Bianca Maria Platania, Salvatore Salomone, Gian Marco Leggio, Filippo Drago, Claudio Bucolo

There were errors in Tables 3 and 4. The correct tables can be found here: 

**Figure:**
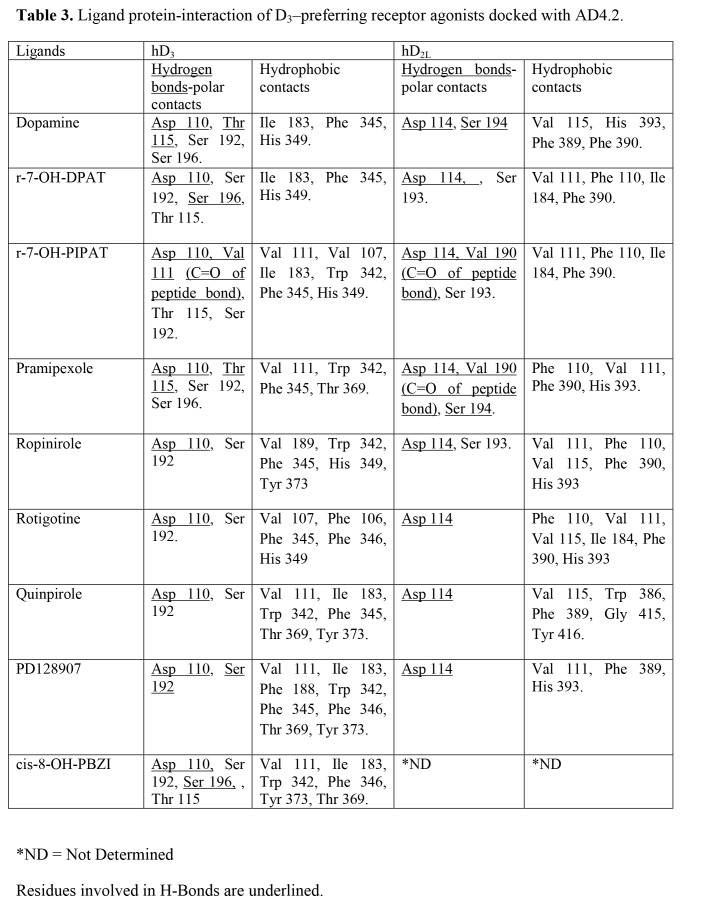


**Figure:**