# Supplementary material for: Homology Modeling of Dopamine D2 and D3 Receptors: Molecular Dynamics Refinement and Docking Evaluation
Source: PLoS One. 2012 Sep 6;7(9):e44316. doi: 10.1371/journal.pone.0044316 (PMC3435408; doi:10.1371/journal.pone.0044316)

**Ki calculation from the Binding Energy of poses generated by Autodock 4.2; source:** http://autodock.scripps.edu/faqs-help/faq/how-autodock-4-converts-binding-energy-kcal-mol-into-ki.

    // equilibrium:   E  +  I  <=>    EI
    // binding:       E  +  I   ->    EI         K(binding),      Kb
    // dissociation:     EI     ->  E  +  I      K(dissociation), Kd
    //
    //                            1
    //         K(binding) = ---------------
    //                      K(dissociation)
    // so:
    //      ln K(binding) = -ln K(dissociation)
    //              ln Kb = -ln Kd
    // Ki = dissociation constant of the enzyme-inhibitor complex = Kd
    //      [E][I]
    // Ki = ------
    //       [EI]
    // so:
    //              ln Kb = -ln Ki
    // deltaG(binding)    = -R*T*ln Kb
    // deltaG(inhibition) =  R*T*ln Ki
    //
    // Binding and Inhibition occur in opposite directions, so we
    // lose the minus-sign:  deltaG = R*T*lnKi,  _not_ -R*T*lnKi
    // => deltaG/(R*T) = lnKi
    // => Ki = exp(deltaG/(R*T))

**Figure S1. Energy plots of systems.** Potential energy (E_pot_) and total energy (E_tot_), of hD_2L_ and hD_3_ receptors.


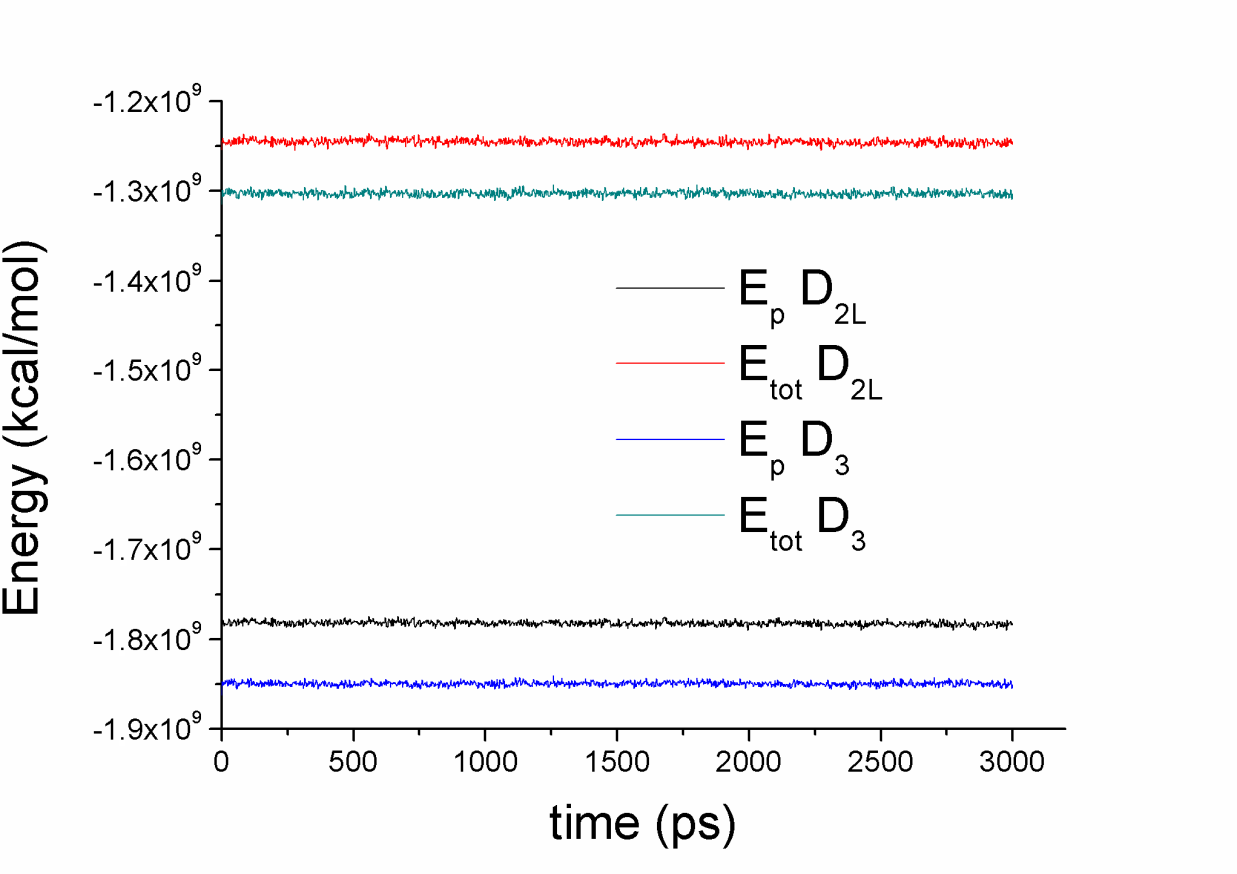


**Table S1. Cα deviations of transmembrane helices (TM) of D_3_ and D_2L_ simulated receptors from the starting models.** Cα deviation values were determined by structural alignment of each helix of the model and of the optimized structure.

| TM helices | D_3_-R  Cα deviation (Å) | D_2L_-R  Cα deviation (Å) |
| --- | --- | --- |
| I | 0.486 | 0.688 ^*^ |
| II | 0.523 | 0.903 ^*^ |
| III | 0.501 | 0.722 ^*^ |
| IV | 0.491 | 0.841 ^*^ |
| V | 0.672 | 1.213 ^*/**^ |
| VI | 0.740 | 0.855 ^*/**^ |
| VII | 0.685 | 0.877 ^*/**^ |

* Major transversal deviation in the intracellular side.

** Major transversal deviation in the extracellular side.

*/** Major transversal deviation in the intracellular and extracellular side.

**Figure S2. Deviation of helices of optimized hD_2L_ receptor (cyan cartoon) respect the starting model (yellow cartoon).** The upper side of the figure corresponds to the extracellular side.


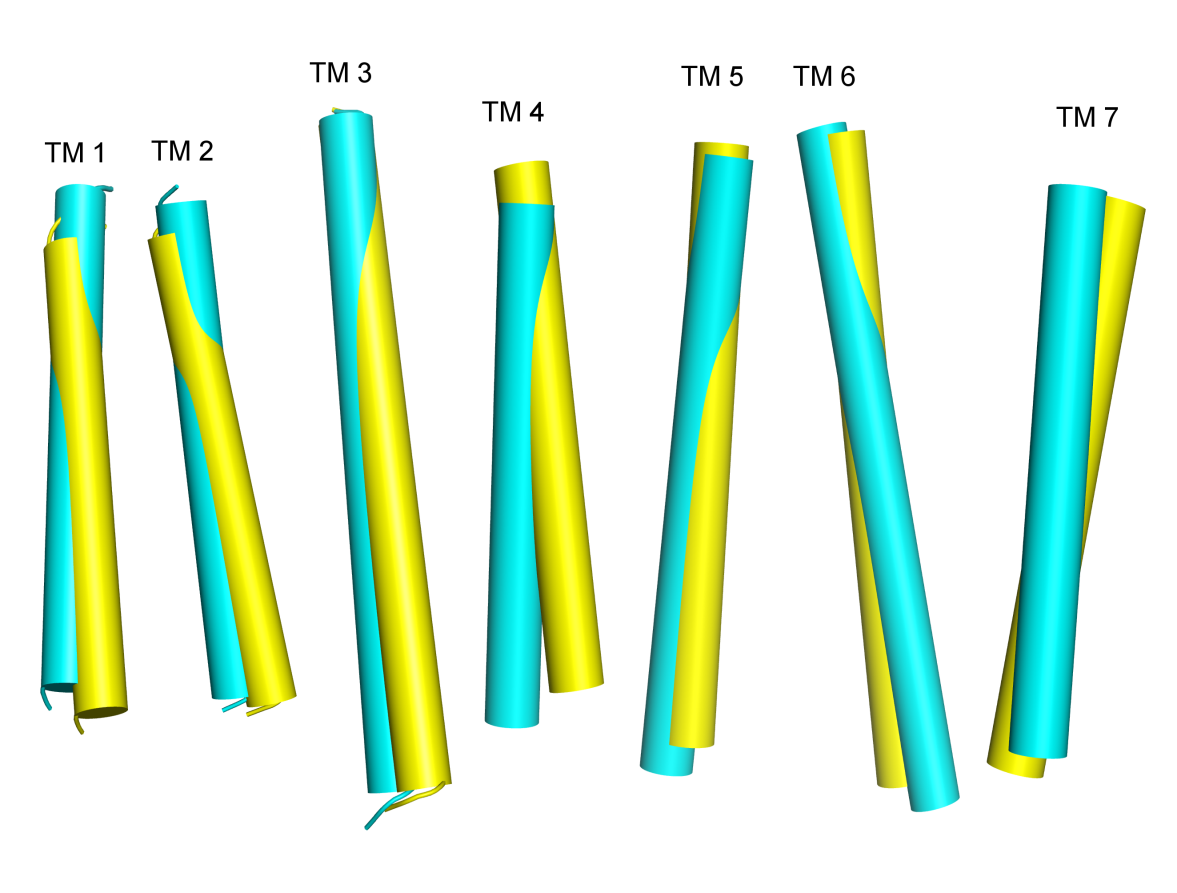


**Table S2. Computed pK_i_ for ligands docked into hD3 and hD2L receptors.** Values are reported for ligands inserted in the regressions represented in Figure 7.

| D_3_ agonist | hD_3_  pK_i_ | hD_2_  pK_i_ |
| --- | --- | --- |
| Dopamine | 4.73 | 4.42 |
| r-7-OH-DPAT | 5.63 | 4.71 |
| r-7-OH-PIPAT | 6.13 | 5.32 |
| Pramipexole | 5.23 | 4.8 |
| Ropinirole | 5.11 | 4.72 |
| Rotigotine | 6.12 | 5.44 |
| PD 128907 | 5.64 | 4.4 |
| cis-8-OH-PBZI | 5.2 | ND |

**Virtual screening results**: the 30 top scored compounds (ZINC-db code) docked into hD_3_ and hD_2L_. The lables _1 and _2 corresponds to different protonation states of ligands.

hD_3_: ZINC45254546, ZINC11847498, ZINC11847496, ZINC04138713, ZINC04262922, ZINC11847499, ZINC04138711, ZINC11847497, ZINC04262921, ZINC04138712, ZINC65739972_1, ZINC04138714, ZINC65739975_1, ZINC65739975, ZINC65739976_1, ZINC63772249, ZINC29554524, ZINC29554520_1, ZINC65739972, ZINC65739978, ZINC63772250, ZINC65739976, ZINC29554520, ZINC29554520_2, ZINC27555141, ZINC27555141_1, ZINC00553652_1.

hD_2L_: ZINC04138714, ZINC04138712, ZINC45254546, ZINC63772250, ZINC11847497, ZINC04262921, ZINC11847496, ZINC04138711, ZINC04262922, ZINC11847498, ZINC63772249, ZINC29554524, ZINC65739978, ZINC65739978_1, ZINC65739976_1, ZINC29554524_1, ZINC29554520, ZINC11847499, ZINC29554524_2, ZINC04138713, ZINC29554520_2, ZINC29554520_1, ZINC00574128, ZINC65739976, ZINC65739975, ZINC65739975_1, ZINC65739972.

**Figure S3. Superimposition of template (3PBL)-homology model- optimized model of hD_3_ receptor and hD_2L_ receptor.** The template structure (green cartoon) is the A chain of hD_3_ receptor crystal structure (3BPL). The cyan cartoon corresponds to the homology model of hD_3_ receptor, the yellow cartoon corresponds to the homology model of hD_2L_ receptor. The optimized models of hD3 and hD2L receptor are respectively the magenta and orange cartoons.


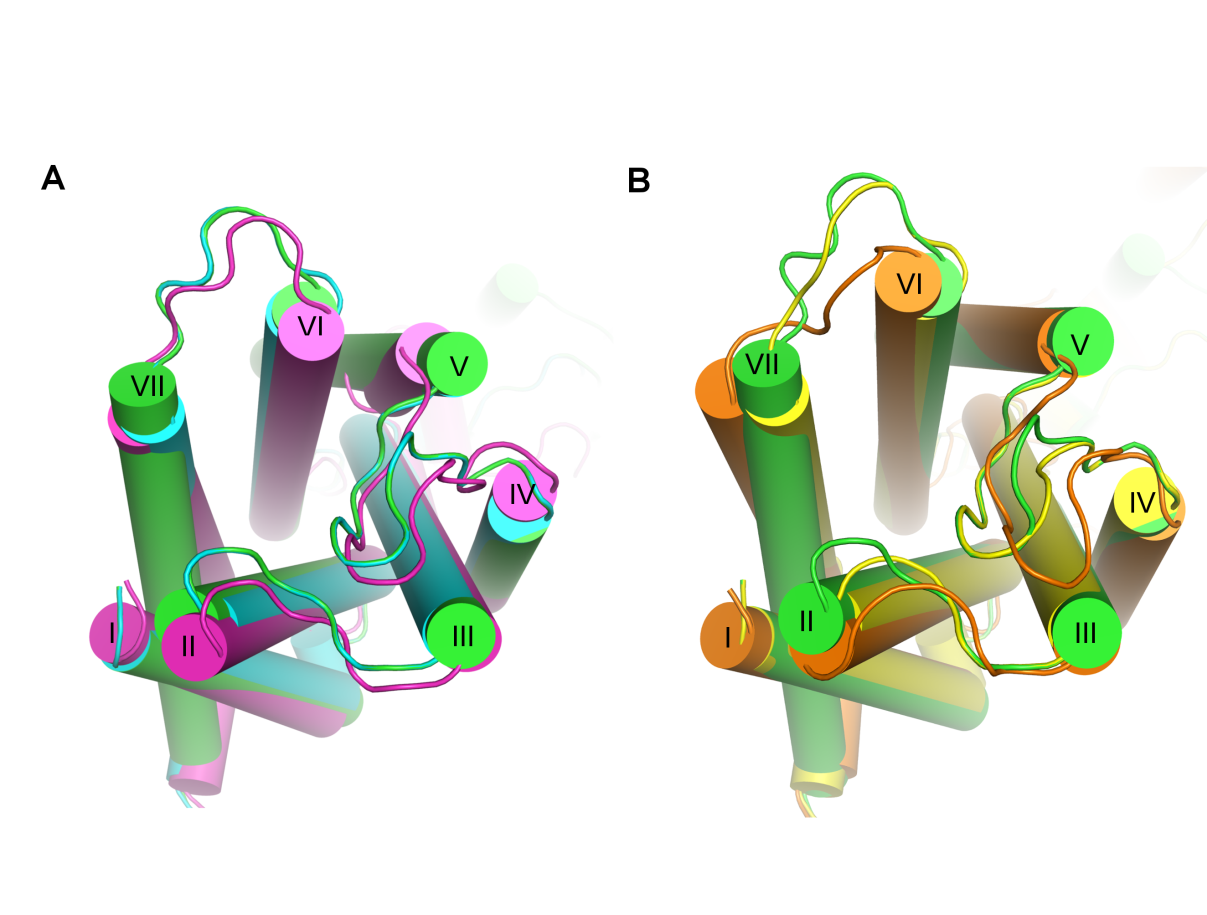

Supplement: Supporting Information S1 — Figure S1: Energy plots of systems. Potential energy (Epot) and total energy (Etot), of hD2L and hD3 receptors. Table S1: Cα deviations of transmembrane helices (TM) of D3 and D2L simulated receptors from the starting models. Cα deviation values were determined by structural alignment of each helix of the model and of the optimized structure. Figure S2: Deviation of helices of optimized hD2L receptor (cyan cartoon) respect the starting model (yellow cartoon). The upper side of the figure corresponds to the extracellular side. Table S2: Computed pKi for ligands docked into hD3 and hD2L receptors. Values are reported for ligands inserted in the regressions represented in Figure 7. Figure S3: Superimposition of template (3PBL)-homology model- optimized model of hD3 receptor and hD2L receptor. The template structure (green cartoon) is the A chain of hD3 receptor crystal structure (3BPL). The cyan cartoon corresponds to the homology model of hD3 receptor, the yellow cartoon corresponds to the homology model of hD2L receptor. The optimized models of hD3 and hD2L receptor are respectively the magenta and orange cartoons. (DOCX) [file pone.0044316.s001.docx]
